# Parasitic Helminths Associated with Two Species of *Serrasalmus* Lacepède, 1803 in the Neotropical Region

**DOI:** 10.1007/s11686-026-01376-0

**Published:** 2026-08-24

**Authors:** Isabela Sales Quagliato, Wagner Toshio Hasuike, Reinaldo José da Silva, Daniel Rodrigues Blanco, Ricardo Massato Takemoto, Heleno Brandão

**Affiliations:** 1 Programa de Pós-Graduação em Recursos Naturais e Sustentabilidade, Universidade Tecnológica Federal do Paraná (UTFPR), Prolongamento da Rua Cerejeira, s/n Bairro - São Luiz, Santa Helena, PR 85892-000 Brazil; 2 Programa de Pós-Graduação em Biologia Comparada, Universidade Estadual de Maringá (UEM), Maringá, PR Brazil; 3 Instituto de Biociências, Setor de Parasitologia, Universidade Estadual Paulista (UNESP), Botucatu, SP Brazil

**Keywords:** Protected area, *Serrasalmus maculatus*, *Serrasalmus marginatus*, South America

## Abstract

**Objectives:**

This study investigated the helminth fauna of *Serrasalmus maculatus* and *Serrasalmus marginatus* in a protected area (Santa Helena Biological Refuge) and in the lower São Francisco Falso River, Brazil. The main objective was to compare the parasitic helminth fauna of these species between the areas of occurrence and to develop an updated list of the recorded parasite species.

**Methods:**

Fifty individuals of each host species were collected between November 2018 and November 2019 using gillnets.

**Results:**

Twelve parasitic taxa were identified, two of which were recorded for the first time: *Spinoxyuris* sp. in *S. marginatus* and *Raphidascaris* sp. (larvae) in *S. maculatus*. The most abundant parasite species in both hosts and sampling areas were *Austrodiplostomum compactum* metacercariae and *Rhinoxenus arietinus*. The helminth fauna differed among the hosts, with *S. marginatus* parasitized with Philometridae gen. et sp., *Spinoxyuris* sp., Digenea gen. et sp.1 and et sp. 2, while *S. maculatus* hosted *Ichthyouris brasiliensis*, *Raphidascaris* sp., and exclusively *Anacanthorus* sp., *Notothecium* sp., and *Rhinoxenus euryxenus*.

**Conclusion:**

This study provides a revised list of helminths associated with *S. maculatus* and *S. marginatus*, including new records of hosts and geographic regions. Comparative analysis highlights differences in the richness and composition of parasite communities between the two Serrasalmidae species, contributing to the understanding of host-parasite relationships in Neotropical freshwater ecosystems.

## Introduction

The Neotropical region contains most of the planet’s freshwater and is characterized by high diversity of freshwater fishes worldwide [7, 45, 46], with an estimated 19,192 species [15]. Aquatic ecosystems in Brazil are home to at least 3780 species of freshwater fishes, and most of this diversity is found in the Amazon and Paraná basins [16]. Besides, fishes host an enormous quantity and variety of parasites, greater than any other group of vertebrates [53], and some of these parasites have medical importance [41].

This study focuses on the helminth fauna of *Serrasalmus maculatus* Kner, 1858 (syn. *S. spilopleura* Kner, 1860) and *Serrasalmus marginatus* Valenciennes, 1837, two “piranha” species that occur in South America [36]. The family Serrasalmidae currently comprises 29 genera and 103 valid species [15, 55].

The comparison of the parasitic communities of the *Serrasalmus* species in this study may aid in identifying infection patterns associated with the hosts’ biological and ecological characteristics [47]. These species share the same environment; therefore, with a partial overlap in feeding habits, they experience similar exposure to parasitic agents, enabling a consistent comparative analysis. Investigating the differences and similarities in the parasitic communities of these hosts contributes to understanding the factors that structure parasite-host interactions in Neotropical environments and may also be useful for future studies.

In this context, it is important to note that *S. marginatus* is considered non-native in the upper Paraná River floodplain, where it has contributed to the decline of native *S. maculatus* populations [39, 61]. It is regarded as native in the lower Paraná River basin, including the present study area [42, 46]. Both species are non-migratory, exhibit external fertilization with some parental care, and are opportunistic predators that feed primarily on fins, scales, and muscle fragments of other fishes [42]. They are also well adapted to lentic environments such as reservoirs and hydroelectric impoundments, where they often become abundant [57].

The selection of these hosts was based on their native status in the study region, ecological relevance, and occurrence in a protected area. Such environments are critical for biodiversity conservation, and parasitological data contribute to ecological monitoring and environmental management.

To date, *S. maculatus* has been reported as host to 16 species of Monopisthocotyla at the species level and one genus, distributed across seven genera [10, 12, 23, 29, 34, 38, 48, 50, 51, 52], two species of Digenea [40, 52], one species of Cestoda [52], three species level and four genera of Nematoda, and one genera of Acanthocephala [10, 52] (For more details, see Table 4).

For *S. marginatus*, there are five species distributed across four genera of Monopisthocotyla [8, 10, 38, 48, 51, 52], two genera of Digenea [51, 52, 56], two species and eight genera of Nematoda [10, 22, 52, 56], and one genera of Acanthocephala [10] have been recorded (For more details, see Table 4).

Given the scarcity of ichthyoparasitological studies in the Area of Relevant Ecological Interest and its main tributary, the São Francisco Falso River, this study aims to provide new parasitological records and compare the helminth communities of *S. maculatus* and *S. marginatus*. Although we did not obtain physicochemical data from the environments, we hypothesize that the composition and richness of helminth assemblages differ between host species due to variations in food availability, habitat use, and feeding behavior of these fish, factors that may influence exposure to intermediate hosts and infective stages, even within a shared environment.

## Materials and Methods

### Study Area and Host Collection

The “Refúgio Biológico Santa Helena” (RBSH) was created in 1984 [19], established and classified as an Area of Relevant Ecological Interest (ARIE) by the National System of Nature Conservation Units – SNUC. The ARIE, which is a remnant habitat of ‘Very High’ priority on the ‘Strategic Areas for Biodiversity Conservation and Restoration’ platform, must be preserved to maintain biodiversity in the state of Paraná, as mandated by the Instituto Água e Terra – IAT (Water and Land Institute) [18].

The São Francisco Falso River is within the ARIE’s area of influence, given its substantial contribution to the formation of the Itaipu reservoir and its status as one of the largest floodplains in the municipality of Santa Helena, state of Paraná [58].

Fishes were collected quarterly between November 2018 and November 2019. This study was authorized by ICMBIO (Chico Mendes Institute for Biodiversity Conservation) via SISBIO (Biodiversity Authorization and Information System) under Permit #57,181, and by the Ethics Committee on Animal Use (CEUA) of the Federal University of Technology of Paraná (UTFPR) under Protocol #2016-031. It was registered at the National System for Management of Genetic Heritage and Associated Traditional Knowledge (SisGen) under Code #AC8FCF0.

**Fig. 1:**
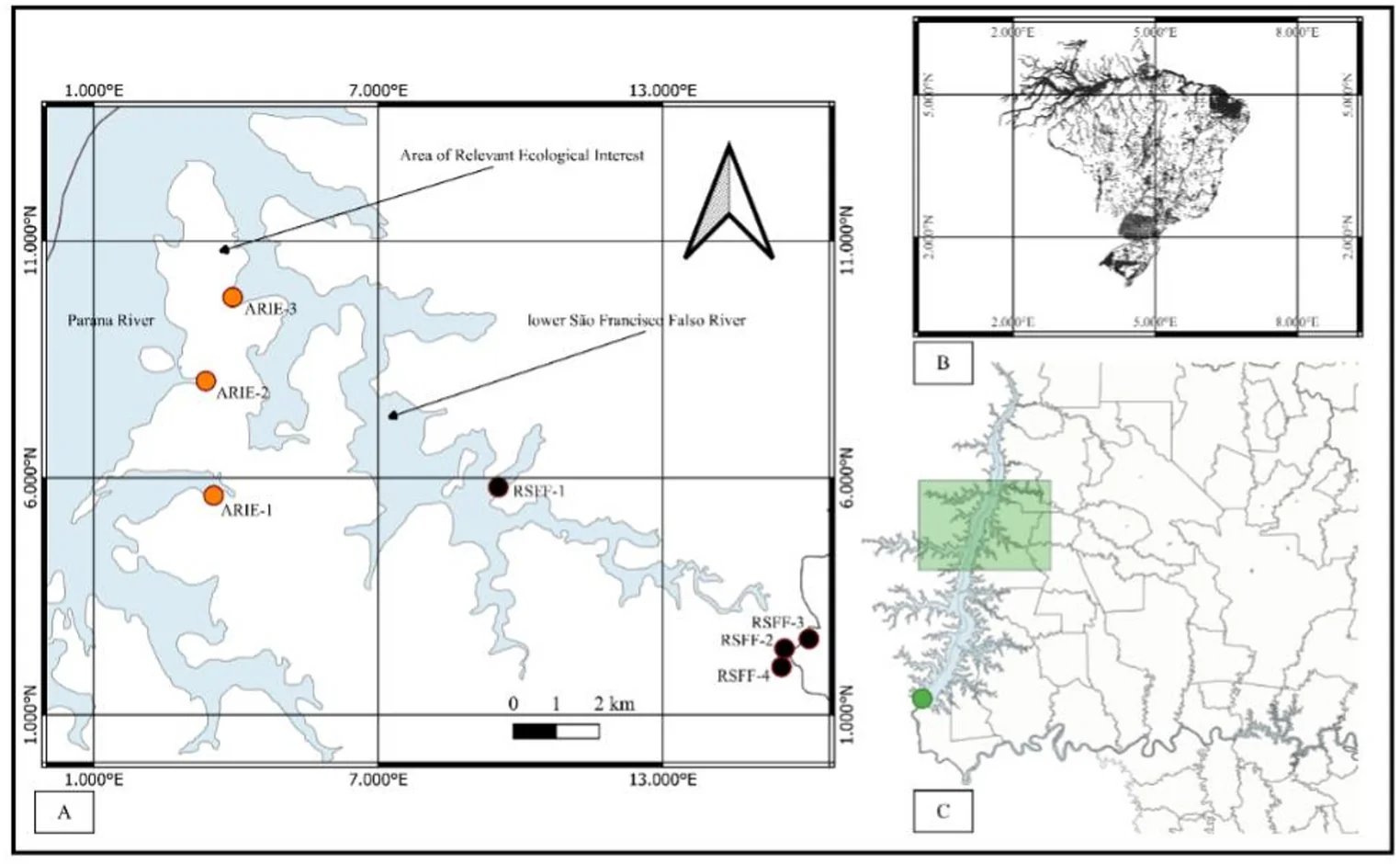
Map showing the distribution of sampling points in the buffer zone (around) of the Area of Relevant Ecological Interest, and along the course of the São Francisco Falso River. **A** distribution of points, where ARIE = Area of Relevant Ecological Interest (orange circle) and RSFF + São Francisco Falso River (Red circle); **B** map of Brazil with emphasis on the state of Paraná, and **C** overview of the study approximate region (green rectangle) and position of the Itaipu dam (green circle). *Source* (QGIS Geographic Information System. Open-Source Geospatial Foundation Project. http://qgis.org”; Google Earth website. http://earth.google.com/, 2020)

A total of 50 specimens of *S. maculatus* and 50 of *S. marginatus* from two different ecosystems was selected for the study, as follows: (1) around the Area of Relevant Ecological Interest (ARIE), located in the municipality of Santa Helena, state of Paraná, and (2) its main tributary, the lower Rio São Francisco Falso (RSFF) (Fig. 1 and Table 1).

**Table 1:** Number of hosts collected per sampling point in the buffer zone (surroundings) of the area of relevant ecological interest, where ARIE = area of relevant ecological interest, and along the course of the São Francisco Falso River, where RSFF = Rio São Francisco Falso - RSFF

Collection points	Latitude	Altitude	collection date	*S. maculatus*	*S. marginatus*
ARIE1 - Semilentic	24°51′15.12′′S	54°21′21.12′′W	03/2018; 06,11/2019	25	15
ARIE2 - Semilentic	24°49′39.97′′S	54°21′27.63′′W	06,11/2018; 06,07,11/2019	7	9
ARIE3 - Semilentic	24°48′30.50′′S	54°21′5.33′′W	03/11/2018; 11/2019	9	9
RSFF1 -Transition	24°51′41.90′′S	54°17′18.50′′W	11/2018; 03,06/2019	2	2
RSFF2 - Lotic	24°53′18.56′′S	54°13′30.32′′W	03/2018; 06,11/2019	1	3
RSFF3 - Lentic	24°53′14.14′′S	54°13′6.60′′W	07/11/2019	3	6
RSFF4 - Lentic	24°53′53.84′′S	54°13′15.12′′W	03/2018; 03,06/2019	3	6
Total				50	50

The fish specimens collected were euthanized, identified, weighed (in grams), and measured (standard length in centimeters) in the laboratory. They were then stored in properly identified plastic bags and frozen at UTFPR’s Ichthyology laboratory on the Santa Helena Campus for further parasitological study.

### Parasitological Analysis

The collected helminths were processed following the parasitological techniques proposed by Eiras et al. [14]. The parasites were identified using an Olympus BX53 microscope. The skin and fins were examined for ectoparasites and encysted helminths. The nostrils were washed with water to collect helminths, and the collected material was examined under a stereomicroscope. Parasites of Monopisthocotyla found in the skin, nostrils, and gills were fixed in 70% ethanol. Some individuals of Monopisthocotyla were mounted in Gray & Wess medium to study their sclerotized structures (anchors, bars, and hooks of the haptor, and copulatory complex).

Following external examination, a longitudinal incision was made in the ventral surface, and the inner organs were removed and placed separately in Petri dishes. The visceral cavity and organs were examined under a stereomicroscope. The collected parasites were fixed in 70% ethanol and stored in vials for future taxonomic procedures. For taxonomical identification, nematodes were cleared with lactic acid using a temporary slide, while trematodes were stained with hydrochloric carmine and cleared with eugenol.

Taxonomic identification of parasites was based on Cohen et al. [11] to Monopisthocotyla, Kohn et al. [21] to Digenea, and Moravec [30, 32, 33] to Nematoda.

Voucher specimens of all the collected helminths were deposited in the Helminthological Collection at the Institute of Biosciences (CHIBB), Department of Biodiversity and Biostatistics, Division of Parasitology, at São Paulo State University (UNESP), in the municipality of Botucatu, state of São Paulo.

### Data Analysis

The name of each helminth taxon, the total number of parasites, the site of infection, prevalence, abundance, intensity of infection, and location of host capture were listed whenever possible. The prevalence (%), mean intensity of infection, and mean abundance of each component of the parasite communities were calculated as described by Bush et al. [9].

To compare the helminth fauna composition between two fish species, the data referring to the parasites from both ecosystems were grouped and subjected to Principal Coordinates Analysis (PCoA) [25]. The analysis was conducted based on a dissimilarity matrix calculated using the Bray-Curtis index, appropriate for abundance data.

A Multivariate Permutational Variance Analysis (PERMANOVA) assessed changes in parasite species composition [6]. We performed 999 permutations to verify significance and applied a pairwise PERMANOVA to assess significant differences between the sampling points.

To test the samples’ sufficiency, the species accumulation curve was calculated using the iNEXT package [17]. The ggplot2 package [59] generated the parasite richness graphs. All analyses were performed using the R software [44] and PAST v.4.13.

Spearman’s rank correlation coefficient (Spearman’s rs) is used to verify the relationship between standard length and parasite abundance in *S. maculatus* and *S. marginatus*. We considered the total number of parasites per individual, grouping them by sex, and performed the analysis separately for each species. Differences in body size between species were evaluated using the Mann–Whitney test. The normality of the data was tested using the Shapiro-Wilk test.

To compile the list of parasitic species already recorded in the two target species, a qualitative, exploratory approach was adopted. The procedure consisted of a bibliographic survey based on data previously published in indexed scientific journals. The following keywords were used: “*Serrasalmus maculatus*”, “*Serrasalmus marginatus*”, “*Serrasalmus spilopleura*”, “parasite diversity”, “parasitic fauna”, “parasitas” and “parasites” in the following platforms: Google Scholar (https://scholar.google.com), SciELO (https://www.scielo.org) and Web of Science (https://www.periodicos.capes.gov.br/index.php/acervo/lista-a-z-bases.html). Studies that presented information directly related to parasite records in the fish species under study were selected, without applying formal inclusion or exclusion criteria. The information obtained was systematized in a table.

## Results

The 50 specimens of *S. maculatus* ranged from 2 to 29.5 cm (mean = 18.8 ± standard deviation = 5.6) in standard length and 0.34 to 805.6 g (307.7 ± 221.3) in weight. The specimens of *S. marginatus* had recorded lengths of 2.5 to 26 cm (14.9 ± 5.5), and weights of 0.35 to 577.3 g (145.2 ± 118.8). There were no statistical differences between the standard length and weight of the populations when compared between sampling sites (*p* ≥ 0.05). Details of the population size structure are presented in Table 2.

**Table 2:** Structure of fish populations collected by sampling point in the buffer zone (surroundings) of the area of relevant ecological interest, where ARIE = area of relevant ecological interest); RSFF = Rio São Francisco Falso - RSFF; Ls = standard length; Wt = weight; Min = smallest size; Max = largest size and SD = standard deviation

	*Serrasalmus marginatus*			*Serrasalmus maculatus*		
	*n*	Ls-Min–Max-DP	Wt-Min–Max-DP	*n*	Ls-Min–Max-DP	Wt-Min–max-(média-Desv)
ARIE 1	15	9.0–26.0 (15.1 ± 5.1)	26.4–326.19(137.82 ± 104.69)	25	11.5–27.3(18.05 ± 5.23)	42.14–780.04(275.42 ± 234.51)
ARIE 2	9	3.0–23.0 (13.5 ± 8.2)	0.88–547.33(205.62 ± 181.46)	7	10.5–29(20.67 ± 5.76)	60.95–805.59(419.95 ± 255.53)
ARIE 3	9	10.5–25.0 (16.7 ± 4.98)	35.75–327.45(153.05 ± 105.4)	9	11.5–25.3(18.5 ± 3.96)	26.56–700.07(266.21 ± 186.27)
RSFF1	2	16.2–19.8(18.1 ± 2.33)	192.53–349.97(271.25 ± 111.32)	2	2.0–20.3(24.9 ± 6.5)	297–641.42(469.21 ± 243.54)
RSFF2	3	10.8–21.1(14 ± 5.3)	49.3–230.01(124.94 ± 93.88)	1	27.0	300.1
RSFF3	6	2.5–102 (26.4 ± 37.4)	0.35–293.78(79.45 ± 106.45)	3	2.0–21.0(14.33 ± 10.69)	0.34–464.89(286.96 ± 250.58)
RSFF4	6	14–20.5(16.1 ± 0.94)	72.65–151.79(102.33 ± 32.51)	3	17.0–23.5(20.63 ± 3.31)	197.27–453.96(348.98 ± 134.57)

We found that 78% (39/50) of *S. maculatus* and 82% (41/50) of *S. marginatus* were parasitized with at least one helminth species. In *S. maculatus*, we recorded eight helminths distributed in three taxonomic groups. For Nematoda, we identified two species, *Procamallanus* (*Spirocamallanus*) *inopinatus* Travassos, Artigas & Pereira, 1928 and *Ichthyouris brasiliensis* Moravec, Kohn & Fernandes, 1992, and one taxon identified only at the genus level, *Raphidascaris* sp.; for Trematoda, we recorded one species, *Austrodiplostomum compactum* Lutz, 1928 (metacercariae); and for Monopisthocotyla, we recorded two species, *Rhinoxenus euryxenus* Domingues & Boeger, 2005, and *Rhinoxenus arietnus* Kritsky, Boeger & Thatcher, 1988, and two taxa identified only at the genus level, *Anacanthorus* sp., and *Notothecium* sp. In total, 1070 parasite specimens were collected, including 951 endoparasites and 119 ectoparasites.

In *S. marginatus*, the helminth fauna consisted of seven taxa distributed in three taxonomic groups. For Nematoda, we recorded one species identified at the specific level, *Procamallanus* (*Spirocamallanus*) *inopinatus* Travassos, Artigas & Pereira, 1928, and one taxon identified only at the genus level, *Spinoxyuris* sp., and one taxon identified at the family level, Philometridae gen. et sp. for Trematoda, we recorded one species identified at the specific level, *A. compactum* (metacercariae), and two taxa identified only at the subclass level, Digenea gen. et sp.1 and Digenea gen. et sp.2; and for Monopisthocotyla, we recorded one species, *Rhinoxenus arietnus* Kritsky, Boeger & Thatcher, 1988, and one taxon identified only at the genus level, *Anacanthorus* sp. In total, 796 parasite specimens were collected, including 691 endoparasites and 105 ectoparasites.

In both host species, considering the total study area, nematodes and trematodes were the most abundant parasites, with a higher prevalence for *A. compactum*. For *S. maculatus*, the most abundant parasite was *I. brasiliensis* (*n* = 464), followed by *A. compactum* (*n* = 365), and for *S. margintus*, *Spinoxyuris* sp. (*n* = 444) followed by *A. compactum* (*n* = 176) (Table 3).

**Table 3:** Parasite species in *Serrasalmus maculatus* and *Serrasalmus marginatus* in the influence of the area of relevant ecological interest, Paraná state, Brazil

Serrasalmus maculatus Kner, 1858	CHIBB	SI	A	MA + SE	MII + SE	*P* (%)
Monopisthocotyla Brabec, Salomaki, Kolísko, Scholz & Kuchta, 2023						
*Anacanthorus* sp.	674 L	gills	5	0.1 ± 0,05	1.25 ± 0.2	6
*Notothecium* sp.	676 L	gills	1			2
*Rhinoxenus arietnus* Kritsky, Boeger & Thatcher, 1988	668 L	nasal cavity	27	0.5 ± 0.3	5.4 ± 2.9	10
*Rhinoxenus euryxenus* Domingues & Boeger, 2005	670 L	nasal cavity	54	1.08 ± 0.5	9.0 ± 2.8	12
Digenea Carus, 1863						
*A. compactum* (Lutz, 1928) metacercariae	673 L	eyes	365	7.3 ± 2.9	14.6 ± 5.8	50
Nematoda Rudolphi, 1808						
*Ichthyouris brasiliensis* Moravec, Kohn & Fernandes, 1992	9206	intestine	464	9.2 ± 4.9	63.3 ± 27.9	14
*Procamallanus* (*S.*) *inopinatus* Travassos, Artigas and Pereira, 1928	9205	Intestine and stomach	49	0.98 ± 2.5	9.8 ± 3.23	10
*Raphidascaris* sp.	9207	intestine	73	1.4 ± 8.4	12.2 ± 9.5	12
*Serrasalmus marginatus* Valenciennes, 1837						
Monopisthocotyla Brabec, Salomaki, Kolísko, Scholz & Kuchta, 2023						
* Rhinoxenus arietnus*	669 L	nasal cavity	75	1.5 ± 0.5	6.8 ± 2.0	22
Digenea Carus, 1863						
* A. compactum* (Lutz, 1928)	666 L	eyes	176	3.5 ± 0.8	12.6 ± 0.7	28
Digenea gen. et sp.1	664 L	intestine	73	1.4 ± 1.2	36.5 ± 23.5	4
Digenea gen. et sp.2	665 L	intestine	15	0.3 ± 0.2	7.5 ± 2.5	4
Nematoda Rudolphi, 1808						
Philometridae gen. et sp.	9203	Intestine and stomach	19	0.38 ± 1.52	3.8 ± 1.68	10
*Procamallanus* (*S.*) *inopinatus* Travassos, Artigas & Pereira, 1928	9204	Intestine and stomach	10	0.2 ± 0.54	2.0 ± 0.62	8
*Spinoxyuris* sp.	9202	Intestine and stomach	458	9.16 ± 10.21	35.23 ± 11.4	26

SI, sites of infection; A, abundance; MA, mean abundance; SE, standard error; MII, mean intensity of infection; P%, prevalence

We identified the following exclusive parasitic species among the analyzed hosts: *Anacanthorus* sp., *I. brasiliensis*, *Raphidascaris* sp., *R. euryxenus*, and *Notothecium* sp., associated with *S. maculatus*; while Philometridae gen. et sp., *Spinoxyuris* sp., Digenea gen. et sp.1, and Digenea gen. et sp.2 were exclusive to *S. marginatus*.

**Fig. 2:**
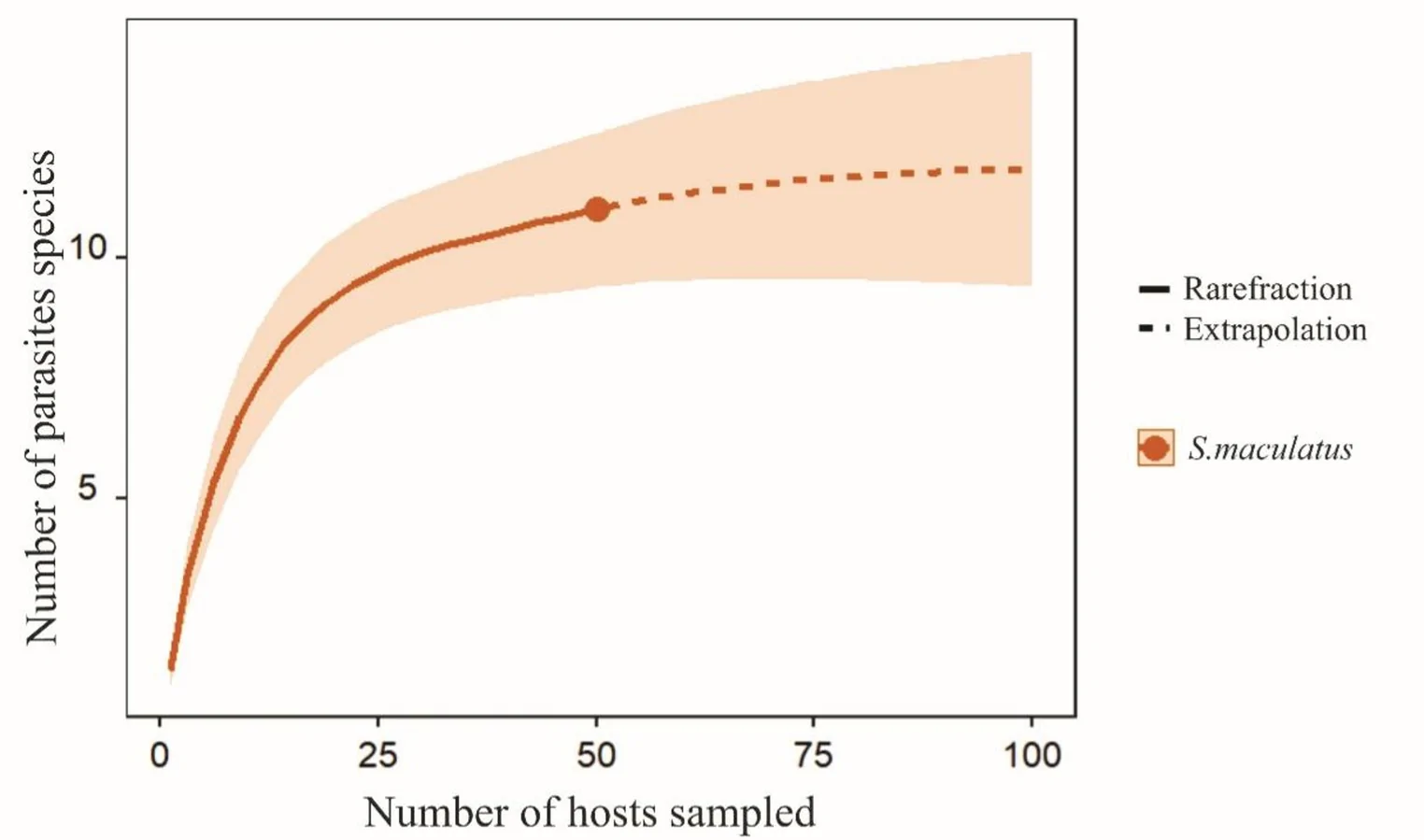
Accumulation curve of parasite species recovered from *S. maculatus*, considering the sum of individuals from the two ecosystems (ARIE and RSFF) sampled

**Fig. 3:**
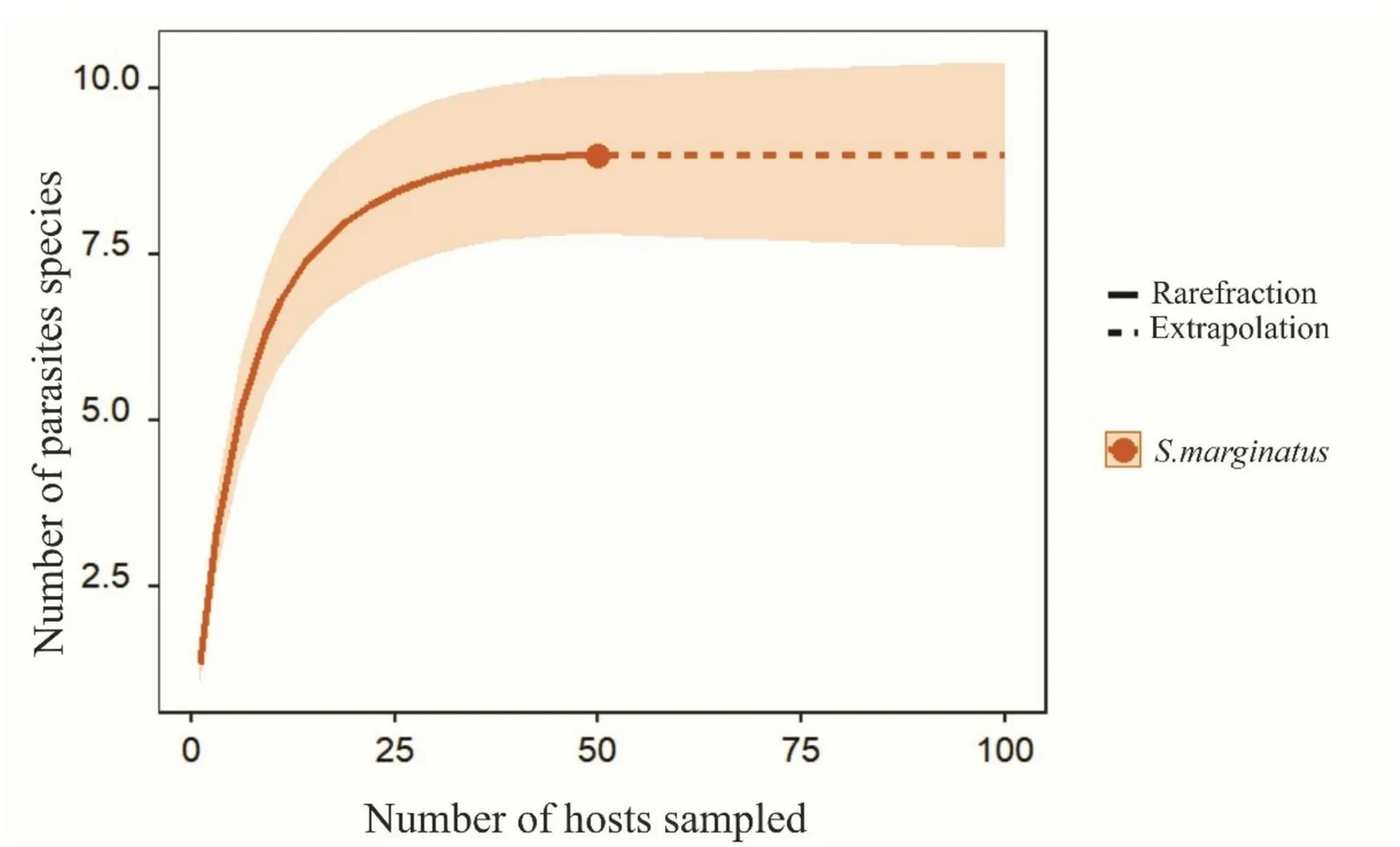
Accumulation curve of parasite species recovered from *S. marginatus*, considering the sum of individuals from the two ecosystems (ARIE and RSFF) sampled

The accumulation curve did not reach the asymptotic level for *S. maculatus*, indicating an even greater parasite species richness, unlike *S. marginatus* (Figs. 2 and 3).

**Fig. 4:**
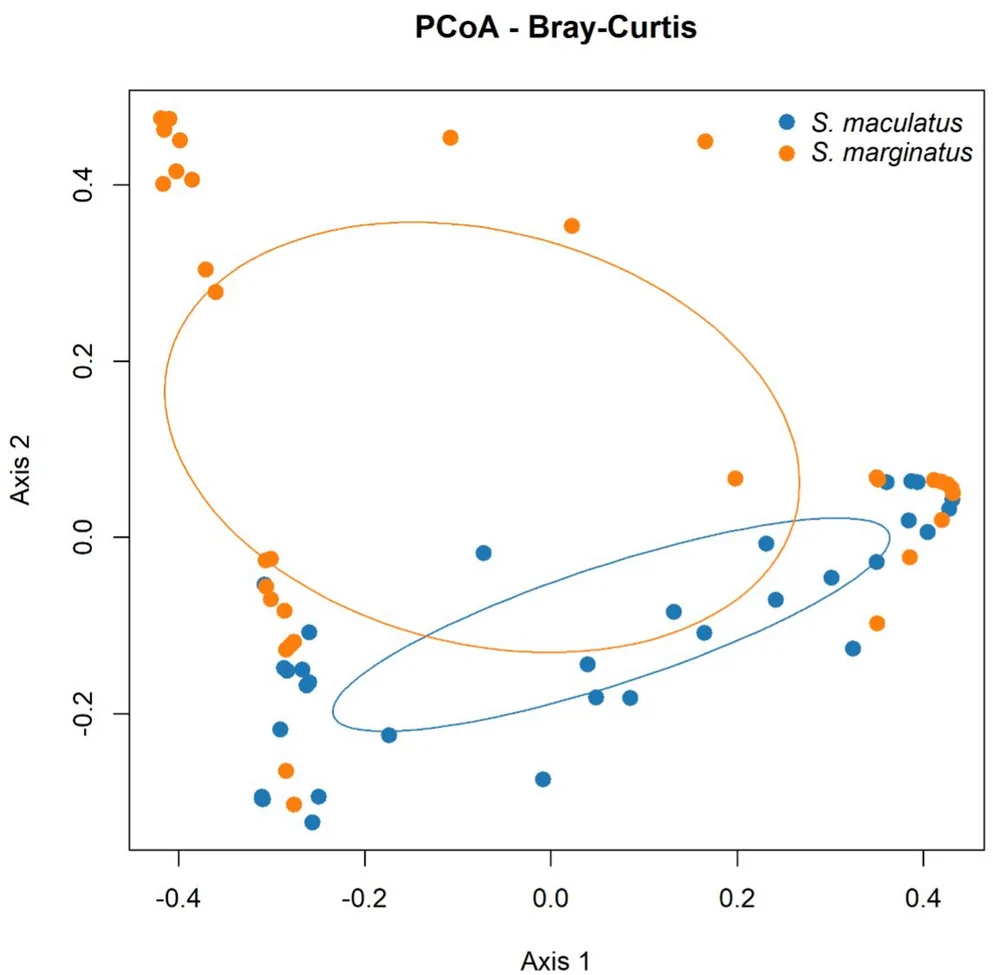
Principal coordinates analysis (PCoA) showing the variability in parasitic composition between *S. maculatus* and *S. marginatus*. PERMANOVA (F = 4.81, R^2^ = 0.063, *p* = 0.001)

Comparing the parasitic fauna, a (dis)similarity in parasite species composition appeared between the verified hosts through a Principal Coordinate Analysis (PCoA) (Fig. 4). A Multivariate Permutational Variance Analysis (PERMANOVA) assessed statistical differences in parasite species composition according to the host species (*p* ≥ 0.05).

Spearman correlation tests indicated a significant association between parasite abundance and standard length only in *S. maculatus* (*p* ≥ 0.05). On the other hand, no significant correlation was observed for *S. marginatus*. Furthermore, *S. marginatus* exhibited a significantly smaller body length compared to *S. maculatus*, as evidenced by the Mann–Whitney test (*p* ≥ 0.05).

To support the results obtained in this study, Table 4 compiles references from the specialized literature that deepen the knowledge about the helminth fauna associated with *S. maculatus* and *S. marginatus*.

**Table 4:** Update of helminthic parasites associated with *Serrasalmus maculatus* and *Serrasalmus marginatus* published in scientific journals

Species list	Locality	Site of infection	Reference
*Serrasalmus maculatus* Kner, 1858			
Monopisthocotyla Brabec, Salomaki, Kolísko, Scholz & Kuchta, 2023			
*Rhinoxenus euryxenus* Domingues & Boeger, 2005	Paraná River basin	Nasal cavity	Osaki-Pereira et al. [38]
*Rhinoxenus paranaensis* Rossin & Timi, 2019	Paraná River basin	Nasal cavity	Osaki-Pereira et al. [38]
*Anacanthorus lepyrophallus* (Kritsky, Boeger & Van Every,1992)	Upper Paraná River floodplain, Paraná state, Brazil; Ilha Solteira reservoir, Grande River, Upper Paraná River basin, São Paulo state, Brazil	Gills	Miguel et al. [29]
*Mymarothecium* sp. Boeger, Piasecki & Sobecka, 2002	Upper Paraná River floodplain, Paraná state, Brazil; Ilha Solteira reservoir, Grande River, Upper Paraná River basin, São Paulo state, Brazil	Gills	Miguel et al. [29]
*Kritskyia annakohnae* Boeger, Tanaka & Pavanelli, 2001	Upper Paraná River floodplain	Uninformed	Takemoto et al. [52]; [10]; Silva et al. [51]
*Amphithecium minutum* Kritsky, Boeger & Jégu, 1997	Amazon River Basin	Uninformed	Kohn and Cohen, [23]
*Amphithecium unguiculum* Kritsky, Boeger & Jégu, 1997	Amazon River Basin	Uninformed	Kohn and Cohen, [23]
*Anacanthorus cladophallus* Van Every & Kritsky, 1992	Amazon River Basin	uninformed	Kohn and Cohen, [23]
*Anacanthorus scapanus* Van Every & Kritsky, 1992	Amazon River Basin	uninformed	Kohn and Cohen, [23]
*Anacanthorus lepyrophallus* Kritsky, Boeger & Van Every, 1992	Upper Paraná River basin, São Paulo state, Brazil	gills	Moreira et al. [34]
*Anacanthorus paraxaniophallus* Moreira, Carneiro, Ruz & Luque, 2019	Miranda River, Pantanal, Mato Grosso do Sul state	gills	Moreira et al. [34]
*Anacanthorus sciponophallus* Van Every & Kritsky, 1992	Batalha River and Peixe River, Upper Paraná River basin, São Paulo state, Brazil	body surface, gills, and nasal cavity	Dias et al. [12]
*Anacanthorus simpliciphallus* Kritsky, Thatcher & Kayton, 1979	Matinha, Maranhão state	gills	Silva [50]
*Mymarothecium perplanum* Kritsky, Boeger, & Jégu, 1996	Amazon River Basin	uninformed	Kohn and Cohen, [23]
*Notothecium modestum* Kritsky, Boeger, & Jégu, 1998	Amazon River Basin	uninformed	Kohn and Cohen, [23]
*Notothecium deleastoideum* Kritsky, Boeger & Jégu, 1998	Peixe River, Upper Paraná River Basin	gills and body surface	Dias et al. [12]
*Notozothecium minus* Boeger & Kritsky, 1988(= *Notozothecium minor* Boeger & Kritsky, 1988)	Batalha River, Upper Paraná River Basin	body surface, gills, and nasal cavity	Dias et al. [12]
*Rhinoxenus piranhus* Kritsky, Boeger & Thatcher, 1988	Paraná River Brazil	nasal cavity	Rossin et al. [48]
*Rhinoxenus euryxenus* Domingues & Boeger, 2005	Paraná River Brazil	nasal cavity	Present study; Rossin et al. [48]
*Rhinoxenus paranaensis* Rossin and Timi 2019	Lower Paraná River (Argentina)	nasal cavity	Rossin et al. [48]
Digenea Carus, 1863			
*Austrodiplostomum compactum* (Lutz, 1928)	Paranapanema River Basin, Lower Paraná Ecoregion	Uninformed	Present study; Yamada et al. [60]
*Prosorhynchus piranhus* Thatcher, 1999	Upper Paraná River floodplain	uninformed	Takemoto et al. [52]
Cestoda Southwell, 1930			
*Proteocephalus serrasalmus* Rego & Pavanelli, 1990	Upper Paraná River floodplain	uninformed	Takemoto et al. [52]; Silva et al. [51]
Nematoda Rudolphi, 1808			
Anisakidae Skrjabin & Karokhin, 1945	Upper Paraná River floodplain	uninformed	Kohn et al. [22]
*Procamallanus* (*Spirocamallanus*) *inopinatus* Travassos, Artigas & Pereira, 1928	Upper Paraná River floodplain, Lower Paraná Ecoregion	pyloric cecum	Present study; Takemoto et al. [52]; [10]; Kohn et al. [22]
*Procamallanus* (*Spirocamallanus*) *neocaballeroi* (Caballero-Deloya, 1977)	Upper Paraná River floodplain	intestine	Casali and Takemoto, [10]
Capillariidae gen. et sp.	Upper Paraná River floodplain		Takemoto et al. [52], Luque et al. (2011)
*Contracaecum* sp. (larva) Railliet & Henry, 1912	Upper Paraná River floodplain	mesentery	Casali and Takemoto, [10]
*Contracaecum* sp. type 1 of Moravec, Kohn & Fernandes, 1993	Upper Paraná River floodplain	mesentery	Casali and Takemoto, [10]
*Contracaecum* sp. type 2 of Moravec, Kohn & Fernandes, 1993	Upper Paraná River floodplain	mesentery	Casali and Takemoto, [10]
*Cucullanus* sp.	Upper Paraná River floodplain	uninformed	Takemoto et al. [52]
*Eustrongylides ignotus* Jäegerskiold, 1909 (larvae)	Upper Paraná River floodplain	uninformed	Takemoto et al. [52]
Philometridae	Upper Paraná River floodplain	uninformed	Takemoto et al. [52]
Acanthocephala Rudolphi, 1808	Upper Paraná River floodplain		Takemoto et al. [52]
*Echinorhynchus* sp.	Upper Paraná River floodplain	intestine and Pyloric cecum	Casali and Takemoto, [10]
*Acanthocephala* gen. et sp.	Upper Paraná River floodplain	Digestive tract	Takemoto et al. [52]; Lehun et al. [26]
*Serrasalmus marginatus* Valenciennes, 1837			
Monopisthocotyla Brabec, Salomaki, Kolísko, Scholz & Kuchta, 2023			
*Rhinoxenus euryxenus* Domingues & Boeger, 2005	Paraná River basin	nasal cavity	Osaki-Pereira et al. [38]
*Rhinoxenus paranaensis* Rossin & Timi 2019	Paraná River basin	nasal cavity	Osaki-Pereira et al. [38]
*Kritskyia annakohnae* Rossin & Timi 2019	Upper Paraná River floodplain	uninformed	Takemoto et al. [52]; Silva et al. [51]
*Rhinoxenus* sp.	Upper Paraná River floodplain	uninformed	Takemoto et al. [52]
*Rhinoxenus arietnus* Kritsky, Boeger & Thatcher 1988	Lower Paraná Ecoregion	nasal cavity	Present study
*Rhinoxenus euryxenus* Domingues & Boeger, 2005	Paraná River Argentina; Paraná River Brazil	nasal cavity	Rossin et al. [48]
*Rhinoxenus piranhus* Kritsky, Boeger & Thatcher, 1988	Paraná River Brazil	uninformed	Rossin et al. [48]
*Anacanthorus* sp.1; sp.2 and sp.3	Upper Paraná River floodplain	uninformed	Takemoto et al. [52]
*Amphithecium* sp.	Upper Paraná River floodplain	uninformed	Takemoto et al. [52]
*Kritskyia annakohnae* Tanaka & Pavanelli, 2001	Upper Paraná River floodplain	urinary bladder	Casali and Takemoto, [10]; Boeger et al. [8]
Digenea Carus, 1863			
*Ascocotyle* sp. (metacercariae)	Upper Paraná River floodplain	uninformed	Takemoto et al. [52]
*Diplostomulum* sp.	Negro River Basin	swimming bladder	Vicentin et al. [56]
Digenea gen. et sp.1	Lower Paraná Ecoregion	intestine	Present study
Digenea gen. et sp.2	Lower Paraná Ecoregion	intestine	Present study
Nematoda Rudolphi, 1808			
*Procamallanus* (*Spirocamallanus*) *inopinatus* Travassos, Artigas & Pereira, 1928	Upper Paraná River floodplain; Negro River Basin	cecum and intestine	Takemoto et al. [52] ; Kohn et al. [22]; Vicentin et al. [56]
*Cucullanus* sp.	Upper Paraná River floodplain	uninformed	Takemoto et al. [52]; Kohn et al. [22]
*Eustrongylides ignotus* Jäegerskiold, 1909 (larvae)	Upper Paraná River floodplain	uninformed	Takemoto et al. [52]
*Goezia* sp. (larva)	Reservoir of the Hydroelectric Power Station of Itaipu, Paraná, Brazil.	uninformed	Kohn et al. [22]
*Contracaecum* sp. (larva) Railliet & Henry, 1912	Upper Paraná River floodplain	mesentery	Casali and Takemoto, [10]; Kohn et al. [22]; Vicentin et al. [56]
*Brevimulticaecum* sp.	Negro River Basin	stomach and mesentery	Vicentin et al. [56]
*Spiroxys* sp.	Upper Paraná River floodplain	mesentery	Casali and Takemoto, [10]
*Hysterothylacium* sp. (larva)	Upper Paraná River floodplain	mesentery	Casali and Takemoto, [10]
*Goezia* sp. (larva)	Upper Paraná River floodplain	mesentery	Casali and Takemoto, [10]
Acanthocephala Rudolphi, 1808			
*Echinorhynchus* sp.	Upper Paraná River floodplain	intestine and Pyloric cecum	Casali and Takemoto, [10]
Acanthocephala gen. et sp.	Upper Paraná River floodplain	Digestive tract	Lehun et al. [26]

Table 5 presents a comparative analysis of the occurrence of parasites in *S. maculatus* and *S. marginatus*, based on records from scientific publications and the data generated in the present investigation.

**Table 5:** Demonstration of the presence and absence of *Serrasalmus maculatus* and *Serrasalmus marginatus* parasites published in scientific journals and the present study

Parasitic species	Serrasalmus maculatus	Serrasalmus marginatus
Monopisthocotyla Brabec, Salomaki, Kolísko, Scholz & Kuchta 2023		
*Amphithecium minutum* Kritsky, Boeger & Jégu, 1997	*	
*Amphithecium* sp.		*
*Amphithecium unguiculum* Kritsky, Boeger & Jégu, 1997	*	
*Anacanthorus cladophallus* Van Every & Kritsky, 1992	*	
*Anacanthorus lepyrophallus* (Kritsky, Boeger & Van Every,1992)	*	
*Anacanthorus lepyrophallus* Kritsky, Boeger & Van Every, 1992	*	
*Anacanthorus paraxaniophallus* Moreira, Carneiro, Ruz & Luque, 2019	*	
*Anacanthorus scapanus* Van Every & Kritsky, 1992	*	
*Anacanthorus sciponophallus* Van Every & Kritsky, 1992	*	
*Anacanthorus simpliciphallus*	*	
*Anacanthorus* sp.1		*
*Anacanthorus* sp.2		*
*Anacanthorus* sp.3		*
*Kritskyia annakohnae* Boeger, Tanaka & Pavanelli, 2001	*	*
*Mymarothecium perplanum* Kritsky, Boeger, & Jégu, 1996	*	
*Mymarothecium* sp.	*	
*Notothecium deleastoideum* Kritsky, Boeger & Jégu, 1998	*	
*Notothecium modestum* Kritsky, Boeger, & Jégu, 1998	*	
*Notozothecium minus* Boeger & Kritsky, 1988(= *Notozothecium minor* Boeger & Kritsky, 1988)	*	
*Rhinoxenus arietnus* Kritsky, Boeger & Thatcher 1988		*
*Rhinoxenus euryxenus* Domingues & Boeger, 2005	*	*
*Rhinoxenus euryxenus* Domingues & Boeger, 2005	*	
*Rhinoxenus paranaensis* Rossin & Timi 2019	*	*
*Rhinoxenus paranaensis* Rossin & Timi 2019	*	
*Rhinoxenus piranhus* Kritsky, Boeger & Thatcher, 1988		*
*Rhinoxenus piranhus* Kritsky, Boeger & Thatcher, 1988	*	
*Rhinoxenus* sp.		*
Digenea Carus, 1863		
*Ascocotyle* sp. (metacercariae)		*
*Austrodiplostomum compactum* (Lutz, 1928)	*	
Digenea gen. et sp.1		*
Digenea gen. et sp.2		*
*Diplostomulum* sp.		*
*Prosorhynchus piranhus* Thatcher, 1999	*	
Cestoda Southwell, 1930		
*Proteocephalus serrasalmus* Rego & Pavanelli, 1990	*	
Nematoda Rudolphi, 1808		
Anisakidae		
*Brevimulticaecum* sp.		*
Capillariidae gen. et sp.	*	
*Contracaecum* sp. (larva) Railliet& Henry, 1912	*	*
*Contracaecum* sp. type 1 of Moravec, Kohn & Fernandes, 1993	*	
*Contracaecum* sp. type 2 of Moravec, Kohn & Fernandes, 1993	*	
*Cucullanus* sp.	*	*
*Eustrongylides ignotus* Jäegerskiold, 1909 (larvae)	*	*
*Goezia* sp. (larva)		*
*Goezia* sp. (larva)		*
*Hysterothylacium* sp. (larva)		*
Philometridae	*	
*Procamallanus* (*Spirocamallanus*) *inopinatus* Travassos, Artigas & Pereira, 1928	*	*
*Procamallanus* (*Spirocamallanus*) *neocaballeroi* (Caballero-Deloya, 1977)	*	
*Spiroxys* sp.		*
Acanthocephala Rudolphi, 1808		
*Echinorhynchus* sp.	*	*
*Acanthocephala* gen. et sp.	*	*

*, presence of parasites

## Discussion

Although both species belong to the same trophic category and share the same ecosystems, our results revealed differences in helminthic composition between them, which may indicate that the ecological characteristics of the hosts (feeding habits, habitat use, body size), geography, and environment can influence the diversity and composition of parasitic communities [3, 27].

Regarding the presence of parasitic species, our knowledge suggests that this is the first record of *Spinoxiurys* sp. parasitizing the gastrointestinal tract of *S. marginatus* and *Raphidascaris* sp. infecting the mesentery of *S. maculatus*, revealing an unequal helminth fauna between the two hosts studied. The species accumulation curve did not reach the asymptote level for *S. maculatus*, suggesting the incidence of an even richer parasitic fauna composition in these locations.

*Spinoxyuris* spp. are relatively rare among freshwater and marine fishes in tropical and subtropical regions [30]. Before this study, they had only been documented in *Metynnis lippincottianus* (Cope, 1870) and *Ageneiosus inermis* (Linnaeus, 1766), as noted by Takemoto et al. [52] and Lehun et al. [26], highlighting the novelty and biogeographical relevance of the present findings. Nematodes of the genus *Raphidascaris* are commonly found in the digestive tract of cichlid fishes and other teleosts [53], and in the Upper Paraná River region they have been recorded parasitizing a variety of host species, including *Prochilodus lineatus* (Valenciennes 1837), *Geophagus brasiliensis* Quoy & Gaimard, 1824, *Geophagus sveni* Lucinda, Lucena & Assis, 2010, *Hypostomus ternetzi* (Boulenger 1895), *A. inermis*, *Loricariichthys platymetopon* Isbrücker & Nijssen, 1979, *Loricariichthys rostratus* Reis & Pereira, 2000, and *M. lippincottianus*. These nematodes exhibit complex life cycles that rely on aquatic environments, both freshwater and marine, utilizing invertebrates and fishes as intermediate or paratenic hosts, and piscivorous birds or mammals as definitive hosts [4, 32, 49]. The present study contributes to knowledge by documenting *S. maculatus* as a new host for *Raphidascaris*, thereby expanding the known host range of this genus in Neotropical freshwater systems.

Another recorded taxon was *Ichthyouris* Inglis, 1962, parasitize freshwater fishes from South America. The first report of this nematode in the Brazilian Amazon was *Ichthyouris ovifilamentosa* Moravec and Thatcher, 2001, parasitizing *Cichlasoma* sp. (Inglis, 1962; Moravec and Thatcher, 2001).

The two species exhibit similar feeding strategies, primarily consuming whole fishes, muscle fragments, and fin portions torn from prey [1, 2]. The availability and abundance of these food resources likely vary across habitats. Such environmental differences in prey composition and accessibility may influence host diet and behavior, thereby contributing to the distinct parasitic profiles observed in this study.

Other ecological and behavioral factors may also contribute to the observed differences in parasitic communities, such as climate (seasonality due to precipitation or temperature), host diversity [28], patterns in parasite diversity associated with host or parasite characteristics [43], differential habitat use, as well as specific behaviors such as territoriality and aggressiveness, which can influence the degree of interaction between individuals and affect the horizontal transmission of parasites. These aspects can modulate the exposure of hosts to different parasitic agents, potentially resulting in distinct patterns of parasite composition. However, for these variables to be considered and quantified, additional studies are needed that integrate more ecological data more comprehensively.

We also observed that *S. maculatus* showed a significant correlation between parasite abundance and body length (rs = − 0,42; *p* = 0,022), while *S. marginarus*, a smaller species, did not show a statistical association between these variables (rs = 0,11; *p* = 0,44). These results suggest that body size may influence parasite load, although this relationship may be modulated by factors such as behavior, degree of parasite aggregation that are influenced by multifactorial phenomena [5], time of exposure to the environment, and availability of resources for the parasites in larger individuals [35] and physiological and behavioral changes [54]. The findings constitute indicators for studies with broader sampling, since the identification of numerical patterns of parasitic dominance is fundamental for understanding the structure of parasite communities. Future research could expand this approach by incorporating host impact assessments.

In comparing the helminth fauna between hosts, we identified parasites of the phylum Nematoda in both hosts, but with distinct species. For *S. marginatus*, we recorded Philometridae gen. et sp. and *Spinoxyuris* sp., while for *S. maculatus*, we observed *I. brasiliensis* and *Raphidascaris* sp. In contrast, only monopisthocotylean parasites were found exclusively in *S. maculatus* (*Anacanthorus* sp., *Notothecium* sp., and *Rhinoxenus euryxenus*), evidencing its monoxenous life cycle. Three taxa were common among both hosts: *A. compactum*, *P.* (*S*.) *inopinatus*, and *R. arietnus*, possibly due to habitat sharing.

Differences in the helminth fauna of these species may also occur in other geographic regions. For example, the populations of *S. maculatus* from the Ilha Solteira hydroelectric reservoir, located in the Upper Paraná River, between the states of São Paulo, Minas Gerais, and Mato Grosso do Sul, Brazil, presented 25 taxa in this host, in which the parasite species *Raphidascaris* sp., *I. brasiliensis*, and *R. arietinus* were not documented [29], suggesting regional differences in their parasite composition. Furthermore, the absence of endoparasites observed by these authors contrasts with the findings of our study.

Among the common parasite species between the two hosts studied, the metacercariae of *A. compactum* constituted a significant infrapopulation. This digenean larva holds ecological significance due to its potential to impair host vision, thereby increasing vulnerability to predation [22]. Its pathological effects may include eyelid inflammation, retinal detachment, lens opacity, and blindness in severe cases [60].

The presence of this parasite in the eyes of regional fish species has been documented for decades. In 1991, it was recorded infecting the ocular tissues of *Plagioscion squamosissimus* Heckel, 1840 in the Itaipu Hydroelectric Power Plant reservoir, as part of a survey involving 32 fish species [20]. More than three decades later, its occurrence persists in the same aquatic system, now observed in two representatives of the family Serrasalmidae examined in the present study. Its continued presence in host species such as *Serrasalmus* spp. indicates that the biological cycle of this parasitic species remains functional in the relationship between intermediate hosts (molluscs and fishes) and definitive hosts (piscivorous birds), integrated into the ecosystem.

In our review of fish parasites in the Upper Paraná River floodplain, *A. compactum* metacercariae were identified in several host species, including *Acestrorhynchus lacustris* (Lütken, 1875), *Auchenipterus osteomystax* (Valenciennes, 1840), *Saxatilia britskii* (Kullander, 1982) (syn. *Crenicichla britskii* Kullander, 1982), *G. sveni*, *Hypostomus regani* (Ihering, 1905), *L. platymetopon*, *Pimelodus ornatus* Kner, 1858, *P. squamosissimus*, and *Schizodon borellii* (Boulenger, 1900) [26]. The present study expands this parasitic distribution by reporting *A. compactum* in the region of influence of the ARIE-SH, further supporting the ecological versatility of this heteroxenous species. Additionally, two digenean species were recovered from the intestinal tract of *S. marginatus* but could not be identified at the species level, underscoring the need for further collections and taxonomic studies to refine their classification.

Among monopisthocotylean infrapopulations observed, we identified relatively common taxa inhabiting the gills and nasal cavities of the host species. The most representative genera recorded were *Anacanthorus* Mizelle & Price, 1965, and *Rhinoxenus* Kritsky, Boeger & Thatcher, 1988. According to Moreira et al. [34], the majority of *Anacanthorus* species are known to parasitize the gills of fishes within the family Serrasalmidae. Notably, *R. arietinus*, abundant in both hosts sampled in this study, has been previously reported infecting the nasal cavities of characiform fishes across the Neotropical region [13, 24].

Nematodes, which are endoparasites that infect fishes in continental, brackish, and marine waters, can cause various diseases in organisms and constitute a significant part of the parasite fauna in different ecosystems around the world [37]. These same authors reported at least 82 freshwater fish species infected with *P.* (*S.*) *inopinatus* in different Brazilian hydrographic regions, which demonstrates, as already reported by Kohn et al. [22], the low degree of host specificity of certain nematodes. The record of this parasite in our study improves the knowledge about its geographic distribution in the Neotropical region.

This study provides relevant information for understanding parasitic diversity in Neotropical fishes by comparing two species of “piranhas” (*S. maculatus* and *S. marginatus*) as hosts of helminths in protected and unprotected environments. The taxonomic characterization of the parasites and the analysis of the parasitic composition in the region contribute to ecological monitoring and the identification of risks in conserved aquatic systems. Knowledge of the biodiversity of parasites and their hosts can assist in assessing environmental health, understanding ecological processes, and guiding wildlife management strategies.

Additionally, our literature review reveals records of parasitic species that appear to be host-specific and other generalists, reinforcing the importance of continued parasitological inventories to better elucidate the geographic distribution and host associations of parasites infecting serrasalmid fishes.

## Conclusion

This study represents the first comprehensive assessment of helminth diversity associated with two species of serrasalmids in the Area of Relevant Ecological Interest (ARIE) and the lower São Francisco Falso River. Larvae of *Raphidascaris* sp. were recorded for the first time in *S. maculatus* and specimens of *Spinoxyuris* sp. in *S. marginatus*, expanding the known geographical distribution of these taxa in the Neotropical region. The results reveal significant differences in parasitic composition between the congeneric species, suggesting that factors such as host species and ecosystem type may influence the structure of helminth communities. Furthermore, the data obtained in protected areas reinforce the potential of parasites as possible ecological indicators, highlighting the relevance of parasitological research in the conservation of aquatic biodiversity.

## Data Availability

The data generated and analyzed during this study are available in the Institutional Repository of UTFPR at the following link: https://repositorio.utfpr.edu.br/jspui/handle/1/28863.
